# Aedes mosquitoes and Zika virus infection: an A to Z of emergence?

**DOI:** 10.1038/emi.2016.37

**Published:** 2016-02-24

**Authors:** Colin R Howard

**Affiliations:** 1 College of Medicine and Dentistry, University of Birmingham, Edgbaston, Birmingham B15 2TT, UK

Once again the world is forced to face an emerging disease threat; this time arising from a mosquito-borne agent that has spread by stealth around the globe.

Zika virus was first discovered in 1947 in the Zika Forest of Uganda during an intensive search for yellow fever virus funded by the British Government and the Rockefeller Foundation. The virus was isolated from both a sentinel rhesus monkey and independently from a pool of *Aedes africanus* mosquitoes,^1^ giving an early indication that Zika virus is essentially a sylvatic infection transmitted from one animal host to another. Humans were long regarded as being the only incidental hosts. Thus, from its discovery until 2007 Zika virus remained an obscure mosquito-borne virus thought to be restricted to a narrow equatorial belt across Africa and Asia. Only 14 or so sporadic laboratory-confirmed cases had been reported until Zika virus spread across the Indian Ocean to South East Asia and Polynesia, with a large epidemic being reported on Yap Island in the Federated States of Micronesia:^2^ nearly 75% of the population were infected. The first indications of the neurological involvement came from the data collected in Polynesia describing Guillain–Barré syndrome and other neurological complications.^3, 4^ Of greater concern, however, is the upsurge in incidence of microencephaly among the new-borns in Brazil over the past year that public health officials believe is linked to Zika virus infection during pregnancy. Although the circumstantial evidence is at first sight convincing, rigorous case-control studies and wider epidemiological studies are needed to confirm this causal linkage.

Initial observations from South America suggest that only one in four adult cases are symptomatic. One issue that needs resolving urgently is an assessment as to the extent of infection in all endemic areas. Such studies are made more difficult, however, as early clinical signs can easily be mistaken for dengue or chikungunya. Human cases present with fever, headache, myalgia and rash, symptoms common to many tropical diseases. Retrospective seroepidemiology studies using virus-specific antibodies must be a priority. As with many emerging diseases, the extent to which a given virus has entered the population affected by an epidemic is often underestimated. For example, there is clear evidence of Ebola virus activity in West Africa prior to the explosive outbreak of 2014.^5^ Where serological studies have been undertaken, it is clear that Zika virus infection has been substantially under-reported. Phylogenetic data from outbreaks in Africa, Asia and Polynesia strongly indicate Zika virus is evolving within human populations: three main genetic lineages have already been identified, one likely originating in Malaysia and being responsible for the 2007 outbreak in Micronesia.^6^ However, the divergence in nucleotide sequence below 12% should allow for the use of Zika virus-specific primers for routine PCR and allow distinction to be made from other flavivirus infections.

As is the case with yellow fever, Zika virus in Africa is most likely maintained in a sylvatic transmission cycle within populations of non-human primates, with humans being incidental hosts. In regions where non-human primates are not available to act as primary reservoirs of infection, humans have become the primary amplification hosts, with the urban mosquito, *Aedes aegypti* being the principle vector for human-to-human transmission. Worryingly the virus also can be transmitted by *Aedes albopictus* the ‘tiger' mosquito, the female of the species feeding silently but aggressively on humans at all times of the day and night. This species is replacing *A. aegypti* in many urban areas and has a much longer life of up to eight weeks. More importantly, adaptation to *A. albopictus* may lead to the emergence of Zika virus strains with a heightened pathogenicity for humans, as has been seen for Chikungunya virus, where a single amino acid change in the E envelope glycoprotein increased virus growth in *A. albopictus*.^7^ We know glycosylation patterns of the Zika E protein are influenced by passage history and this may be a focus of future studies for clinical isolates.

Little is known regarding the pathogenesis of Zika virus infection. There must be a substantial viraemia following the incubation period to allow the ease of human–mosquito–human transmission that is so evident in current and past outbreaks. Whether or not these levels are the same among asymptomatic cases needs to be established before mathematical models of transmission will prove useful. There is some evidence of sexual transmission, not surprising if the viraemia is high, although the risk of travellers indulging in unprotected sex needs to be better defined. High and prolonged virus excretion in urine for at least 10 days after onset presents a further risk for containment. Early laboratory studies show that the virus grows well in cultured skin fibroblasts, and we can expect rapid progress at the molecular level.^8^ How this will translate into treatment and control strategies in the short term remains to be seen, however, work is needed in developing an animal model of teratogenic infection. Unravelling the complex interactions between the mother's infected tissues and foetal development will be an arduous task. It may well be that other co-factors also are important in pregnancy and detailed analyses of risk factors will take time.

It has been widely commented that a Zika vaccine could be developed rapidly, no doubt encouraged by the rapid deployment of experimental vaccines during the recent West African Ebola virus epidemic. But there were already a number of candidate Ebola virus vaccines under development and thus such optimism that a Zika vaccine could be developed within a year are misplaced. It should be remembered that the development of a vaccine against the four serotypes of the serologically related dengue virus has been fraught with difficulties. More encouragingly perhaps, existing vaccine development platforms, for example, chimeric vaccines using yellow fever 17D vaccine as a ‘backbone', have been successfully used for the development of a vaccine against the flavivirus West Nile that entered North America in 1999.^9^ But even if a vaccine becomes available, the largely unpredictable nature of Zika virus emergence and the task of immunising large numbers of people living in endemic zones would mean that mass vaccination would not be cost-effective, especially as presently adult infections are relatively minor or asymptomatic. Perhaps more efficient prevention might be obtained using any Zika vaccine in women of child-bearing age. Caution will be needed, however, to ensure no harmful teratogenic effects result from immunopathogical reactions to Zika virus proteins.

For the present, the control of *A. aegypti* is the most urgent need, all the more so given the ease in which other flaviviruses can spread into indigenous mosquito species and thus promulgate virus spread. Present day public resistance to the use of insecticides needs to be overcome plus more effort given to educating residents in affected areas to cover even the smallest pools of stagnant water. An excellent example of how such control can be implemented is the state of Singapore that pursues an aggressive policy of both inspection and public awareness campaigns. Lessons are also to be had from history: in 1900 the seminal work of Walter Reed and colleagues led to a drastic reduction in the incidence of yellow fever in Cuba once *A. aegypti* had been confirmed as the principal vector. The Panama Canal was only completed once mosquito eradication significantly reduced the risk of disease. As is so often the case, however, a political will to implement effective strategies is imperative.

That Zika virus has essentially followed the expansion of chikungunya around the world should alert us all to other potential risks, such as the potential expansion of other African viruses, for example, the alphavirus O'nyong nyong. Zika virus also brings into the spotlight once more the matter of surveillance. Much of what we know today as to mosquito and tick-borne infections is thanks to extensive surveillance studies undertaken by a small cohort of scientists in the 1950s and 1960s working mainly in the tropics with either government funds or philanthropic support. Sadly, these efforts have largely ceased or reduced into programmes of hypothesis-driven research into known diseases. Surveillance studies are neither fashionable nor popular with those seeking scientific advancement. Yet were countries with the resources prepared to invest in applying modern technologies to better understanding the ecology of diseases and the balance between human advancement and manipulation of the world around us, we would be better prepared when the unexpected emerges.
